# Sonoluminescence from single cavitation bubbles near solid surfaces

**DOI:** 10.1016/j.ultsonch.2026.107815

**Published:** 2026-03-17

**Authors:** Jaka Mur, Rok Petkovsek, Claus-Dieter Ohl

**Affiliations:** a Faculty of Natural Sciences, Institute for Physics, Department Soft Matter, Otto-von-Guericke University Magdeburg, 39106 Magdeburg, Germany; b Faculty of Mechanical Engineering, University of Ljubljana, Askerceva 6, 1000 Ljubljana, Slovenia

**Keywords:** Sonoluminescence, Cavitation, Cavitation erosion, Laser-induced breakdown, Time-resolved imaging

## Abstract

Sonoluminescence, the light emission from bubbles undergoing large volume oscillations, has traditionally been associated with near-spherical bubble collapses in free liquid. In contrast, highly asymmetric bubble collapses near solid boundaries are known to cause surface erosion from transient and extreme pressure build ups. While both phenomena are caused from energy focusing during bubble collapse, a connection between erosion and light emission has not been previously reported. Here, we for the first time observe the highly asymmetric toroidal cavitation bubble collapse leading to erosive effects on the nearby boundary emits light, too. Through multi-modal measurements of the bubble dynamics and the associated phenomena near solid surfaces, we find that light emission occurs exclusively at the sites and time of the most intense flow focusing. Yet, we find no correlation between the occurrence of light emission from sonoluminescence and erosion. This findings reveal that the mechanisms governing energy focusing within the bubble and in the liquid are resulting from distinct energy focusing pathways.

## 1 Introduction

Among the most fascinating and least understood fluid mechanic phenomena is the energy focusing of vapor bubbles in a liquid during their pressure driven shrinkage, recognized more than a century ago [1]. During this so-called cavitation bubble collapse, liquid kinetic energy is focused by up to 12-orders of magnitude [2]. The result is the emission of shock waves [3], [4], [5], [6] and photons [7], [8], [9] when the bubble reaches the minimum volume. Controlled cavitation thus found numerous applications such as recycling [10], cell lysis [11], disinfection [12], metal peening [13], and medical therapy [14], [15].

While controlled energy focusing of collapsing bubbles leads to desirable effects, the same phenomenon causes cavitation erosion. For decades, it has been known that cavitation bubbles driven only by atmospheric pressure erode or deform a metallic boundary when collapsing very close to it [16], [17], i.e., overcome the elastic deformation limit [18], which remains an active research topic [19], [20], [21]. In recent studies, several pathways to surface deformation and erosion were identified, ranging from jets [22], through secondary strong collapse areas [23] to shock wave focusing [24], [25], depending on the relative bubble-to-wall distance and material deformability. The latter mechanism applies to bubbles closest to a solid surface and leads to the strongest erosion. For this case, it was recently revealed how the pressure is amplified [24]: When the bubble shrinks very close to the boundary, it forms a small torus in the last stages just before reaching minimum volume. Inherent asymmetries result in a torus that is collapsing along two specific directions, where the initial shock wave is amplified with the successive collapse of the remaining bubble. At the location where the shock waves finally superimpose, i.e., on the opposite side of the start of the collapse, needle-shaped depressions are formed in the solid surface [24], providing a clear connection between energy focusing and erosion [26], [27].

Sonoluminescence, the light emission accompanying collapses of bubbles undergoing large volume oscillations, is a testimony to the extreme conditions reached within the bubble [28]. Sonoluminescence appears when a collapsing cavitation bubble achieves conditions roughly in the range of: temperatures above few-thousand K, pressures $10^{3}-10^{4}$ higher than the surrounding atmospheric pressure, and short collapse timescales, typically sub-nanosecond at minimum radius [29]. The light emission is explained by the compression and heating of the bubble content [9], [30] that may even form plasma [31] inside. Historically, sonoluminescence was connected with near-spherical bubble shapes and bubbles that collapse in a less spherical fashion have reduced light emission by orders of magnitude or none at all [8], [32]. In particular, transient cavitation bubbles only emit light when they are created sufficiently far from a boundary that would disturb the spherical collapse, i.e., more than three maximum radii away [33], [34]. In the case of bubbles collapsing in a pressure gradient, luminescence ceases already when the bubble is equivalently deformed as it would be 7.5 maximum radii away from the boundary, as reported by Supponen et al. [35]. In summary, existing experiments have consistently shown that the atmospheric-pressure driven bubble collapse must be close to spherical, otherwise the energy focusing is not sufficient to heat the gas to temperatures needed for visual light emission. The emission itself is enhanced in certain water-based solutions [36] or by lipid-coatings keeping the spherical shape [37].

Here we use a multi-modal yet contemporaneous measurements of highly non-spherical bubble collapses near surfaces. This allows to simultaneously observe ultra high-speed bubble dynamics, measure temporally and spatially resolved light emission intensity and shock wave emission, as well as surface erosion on a single bubble basis, in contrast to works relying on cumulative effects [38]. We reveal that the strong energy focusing leads not only to erosion but also luminescence, identifying the mechanisms for this extreme regime of fluid structure interaction.

## 2 Materials and methods

**Fig. 1:**
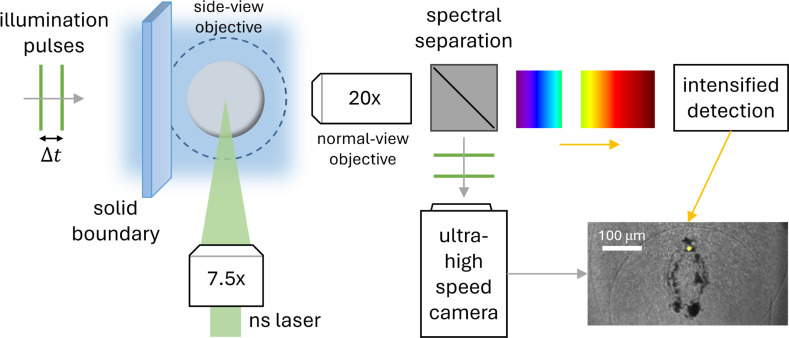
A schematic representation of the experimental setup, depicting crucial experiment parts. By using two spectrally separated cameras in the normal-view direction, the high-speed camera frame closest to the bubble collapse can be precisely overlaid with respective sonoluminescence emission (bottom right).

The experimental setup was based around a laser-generated cavitation bubble near a solid boundary (Fig. 1). The cavitation bubble was formed with a Q-switched green nanosecond laser, working at a 532 nm wavelength with the pulse duration of 6 ns (Litron Nano SG-100-2), coupled to a laboratory made 3D-printed experimental chamber with glass sides and bottom. The emitted laser pulse was focused with a Mitutoyo 7.5× long-working distance microscope objective with a numerical aperture of 0.21. The laser energy was kept constant to achieve stable bubble radii, resulting in bubble lifetimes of 90−100μs near solid boundaries made of either glass, quartz, or polished aluminum.

### 2.1 Imaging

The experimental setup allowed the bubble collapse area to be imaged simultaneously using either a dual- or triple-camera setup. At least one camera was placed in the normal-view for sonoluminescence detection and normal-view bubble dynamics, and one perpendicular to it in the side-view for γ measurement and side-view bubble dynamics. The directions are defined with respect to the solid boundary, schematically presented in Fig. 1. The schematic illustration in Fig. 1 shows an example of using two spectrally separated cameras in the normal-view direction. The output of this setup is presented in the high-speed camera frame closest to the bubble collapse that can be precisely overlaid with respective sonoluminescence emission (bottom right in Fig. 1). The elliptic or horse-shoe bubble shape at the moment of collapse, i.e., at the minimal volume moment, is due to the close boundary proximity of bubble generation. This results in two intense collapse spots, one at bubble top and bottom, similar to other works on cavitation erosion using single laser-induced bubbles [24]. The sonoluminescence emission was detected at the top collapse spot in the presented example in Fig. 1.

Bubble dynamics and emitted shock waves were resolved with a combination of an ultra high-speed camera (Shimadzu Hyper Vision HPV-X2) and laser-based illumination. The pulsed laser-based illumination source allows us to capture the shock waves emitted without motion blurring, as each image is illuminated with laser pulses from a 5  MHz femtosecond laser (Ekspla FemtoLux, 515 nm center wavelength). The laser pulses are split and separated through an optical delay line by 20 ns, so that each frame of the high-speed camera is exposed twice. Multi-illumination imaging enabled us to resolve shock wave propagation at two different times on a single camera frame close to the collapse site, similar to [20], [39], facilitating precise collapse time measurements through shock wave emission timing interpolation. It is possible to deduce the collapse time with a 10-fold improved precision compared to the frame interval due to the illumination. The illumination laser pulses were coupled into a multi-mode optical fiber, while on the output side a combination of a fiber optic collimator and a focusing lens was used to achieve sufficient brightness in the field of view. Band pass and band stop filters for the illumination wavelength of 515nm allowed a spectral separation of the high-speed camera image from the recording of the fainter sonoluminescence emission. After spectral separation of the illumination source, the rest of the captured light was guided to an intensified image detection unit, missing the spectral part in range of 500–550 nm (illustrated in Fig. 1).

*Sonoluminescence intensity measurements* were done using a dual- camera setup. This set of experiments combined the ultra high-speed camera for shadowgraphy-based bubble dynamics recording positioned in side-view with an intensified camera in normal-view. The ultra high-speed camera was set at a 1 MHz framing rate and had a 3.1μm/pixel resolution (as shown in Fig. 2a). The imaging resolution of measurements in side view (Fig. 2) resulted from using a 10x magnification long working distance microscope objective (Edmund Optics, numerical aperture 0.28).

The intensified camera was used to record the faint sonoluminescence emission viewing through a long working distance microscope objective (Edmund Optics, 20× magnification, numerical aperture of 0.42) in normal-view. A single frame ICCD (LaVision IRO system (P43 phosphor screen, S20 photocathode, 2:1 relay optics) ) coupled with a PCO.1600 camera was used for sonoluminescence intensity measurements (resulting final image resolution is 1.75μm/pixel). The perpendicular directions of imaging were chosen as an additional precaution to eliminate any possible illumination light leakage into the intensified imaging branch.

*Time-resolved sonoluminescence and collapse measurements* were done using a triple-camera setup. The ultra high-speed camera was set up to a 5 MHz framing rate (resolution of 400 × 250 pixels) for recording of the collapsing cavitation bubble and emitted shock waves in normal-view. For these measurements the ultra high-speed camera (Fig. 3) was combined with a 20× magnification long working distance microscope objective (Edmund Optics, numerical aperture 0.42, resulting magnification 1.5μm/pixel on the final images) together with the intensified camera.

The intensified camera setup used to record the sonoluminescence emission timing and position consisted of the Photonis Cricket™${}^{2}$ image intensifier (P46 phosphor screen, Hi-QE green photocathode, 1:1 relay optics) coupled with a time-stamping TPX3CAM. The TPX3CAM uses a silicon sensor bonded to Timepix3 integrated circuit (ASIC) with 256 × 256 pixels (final image resolution of 2.75μm/pixel). The signal on each pixel of the 256 × 256 pixel detector is individually compared to a predefined threshold after amplification. If the signal is higher than the threshold, the camera records the photon time of arrival (ToA) as well as the time over threshold (ToT). The latter serves as an estimate of signal amplitude, in our experiments directly related to the number of photons incident on each pixel. The electronics in each pixel processes the incoming signals to measure their ToA for events that cross a predefined threshold with a 1.56 ns precision [40].

This combination of cameras coupled through the same 20× magnification microscope objective enabled simultaneous and spatially overlapped shadowgraphy-based microscopy imaging of the bubble collapse with the corresponding light emission measurement. An example overlaid image is shown in the bottom right of Fig. 1 and further results in Fig. 3.

The third camera, positioned in side-view, was a high-speed camera Photron Fastcam Mini AX200 using 2×–5× magnification objective (Canon MP-E 65 mm f/2.8) to determine the distance of the bubble center from the solid surface. As a secondary high-speed camera it was coupled to a narrow-band continuous LED illumination source in green to avoid disruption of the intensified imaging branch. The third camera is not shown in Fig. 1 for clarity of presentation.

The measurements presented in the *Correlation of sonoluminescence, shock waves, and erosion* Section used in principle the same triple-camera setup, but utilized the ICCD camera described above due to limited equipment availability.

### 2.2 Materials and shock wave monitoring

Single-event erosion recognition and top-view bubble dynamics imaging were done using a 6 mm thick quartz window (Edmund Optics) as a boundary due to its transparency and resilience to structural cracking. The surface topography scan of eroded patterns presented in Fig. 5 in the main text was obtained with a Mahr MarSurf CM Explorer microscope on the quartz substrate. Sonoluminescence intensity measurements were also repeated near polished aluminum and borosilicate glass surfaces. The position of the laser focus with respect to the solid surface was controlled by mechanical movements of the sample and measured with a side-view camera. The collapse shock wave properties were measured using a Mueller-Platte needle-probe hydrophone, enabling us to extract shock wave ToA and energy.

## 3 Results and discussion

**Fig. 2:**
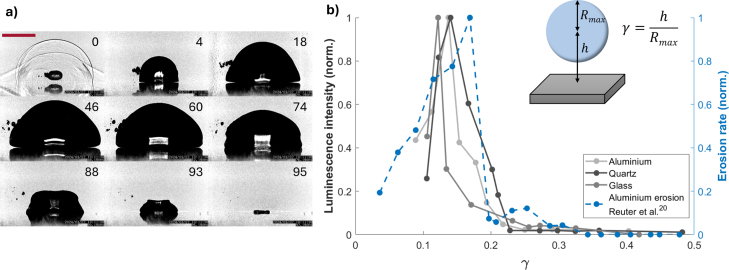
(a) High-speed sequence of the first oscillation of a cavitation bubble generated close to a solid boundary in side view in the cavitation erosion regime. The timestamp t=0 is defined as the frame closest to the bubble generation. Then, the bubble reaches maximum size after 46 μs, and collapses in a toroidal shape at t=95μs. The toroidal shape close to collapse is only visible in normal view, e.g., Fig. 1. The scale bar is 400μm. The whole video is presented in Supplementary Movie 1, and a higher frame rate video of a similar event in Supplementary Movie 2. (b) Normalized light intensity emitted during bubble collapse near a boundary as a function of the non-dimensional distance γ with added erosion rate measurements from [24] for comparison. In inset, representation of the non-dimensional distance parameter $\gamma =h/R_{\max}$ in the experiment, where h is the distance of the laser focus from the boundary, and $R_{\max}$ the bubble radius at maximum expansion, measured perpendicular to the surface.

### 3.1 Sonoluminescence intensity at boundary

Energy focusing during the non-spherical collapse of a single cavitation bubble can be studied conveniently with laser-induced cavitation bubbles. Here, the vapor bubble is nucleated with a focused nanosecond laser pulse, as a result of a highly localized dielectric breakdown in water. After the laser pulse, the bubble expands explosively, reaches its maximum size, and then collapses. A boundary nearby gives rise to asymmetries, preventing a spherical convergent flow and thus a spherical bubble shrinkage or collapse.

An extreme example of near-boundary bubble dynamics as viewed in side-view is presented in Fig. 2a. The bubble was nucleated at a non-dimensional stand-off distance of $\gamma =h/R_{\max}=0.15$. Here, γ is defined as a ratio of dimensional distance h of the laser focus from the boundary, and $R_{\max}$, the bubble radius at maximum expansion, measured perpendicular to the surface, in line with other works [27] and presented graphically in the inset of Fig. 2b. The bubble expands to a nearly hemispherical shape at its maximum expansion, t=46μs, develops a circumferential kink parallel to the boundary during shrinkage visible at t=88μs, and collapses into a toroidal shape at t=95μs.

The light emission measurements are gated to the expected collapse time interval, taking into account 90–100μs first bubble oscillation duration. Bubble collapses very close to the boundary, i.e., at γ<0.3, were found to emit light. Fig. 2b depicts the integrated and normalized light intensity emitted by the collapsing bubbles for the 10 brightest events at each stand-off distance γ, representing one measurement point. The laser-generated bubble is initiated at a given stand-off distance γ from the surface in the range 0<γ<0.5 The sonoluminescence signal reaches a maximum at γ=0.12−0.15 for all measurement series using aluminum, quartz and glass boundaries, falling quickly for closer or farther distances from the boundary. Above γ=0.25, the sonoluminescence is close to the noise level and fell below detection limits for γ≥0.5. We count light detection as a sonoluminescence event if at least one pixel in the observed area has a value 50 % above the background levels and time within the expected time interval close to the collapse. The luminescence signal in Fig. 2b is normalized for each material for better comparison. The peak values on glass were approximately two times higher than on quartz and aluminum, probably due to the excitation of auto-fluorescence, which was directly observed for our glass samples after excitation by the laser-induced plasma. When comparing the light emission from spherical bubble collapses to the brightest events from the non-spherical bubbles, we find about a 100-times lower count as compared to the spherical bubble for the same laser energy seeding the bubble. Even though the initial bubble oscillation duration is similar for bubbles near the boundary and the comparable spherical bubbles, thus also making the total bubble energy comparable, there are significant energy losses present near the surface. The most prominent energy dissipation mechanism can be recognized in the partial toroidal collapse leading to the main point-like collapse and the resulting erosion of the surface. Therefore, a significant amount of bubble energy is converted to shock waves. As shown in previous research, sonoluminescence intensity depends strongly on the bubble shape. Likewise, in the near-boundary scenario, the exact bubble shape in the final stages of the collapse crucially depends on γ and any possible asymmetries in the bubble generation. The variations in coupling of laser energy into bubble formation contribute to small changes in γ, but to important changes in light emission. Not every event results in sonoluminescence, and the sonoluminescence intensity varies by up to two orders of magnitude at the same parameters.

The light emission is strongly sensitive to the distance γ and thus the focusing of the kinetic liquid energy. A similar sensitivity was observed in Reuter et al. [24] for the erosion of an aluminum sample. Their data is shown normalized in Fig. 2c with a dashed line. The similarity between luminescence and erosion suggest a strong correlation between the two consequences of energy focusing, e.g., heating of the bubble interior and the extreme fluid–structure interaction. Next, we study experimentally if there is a causal determination between the two phenomena, i.e., if they are concurrent and coincident.

### 3.2 Time-resolved collapse and sonoluminescence imaging

In order to seek this answer, we replaced the intensified camera with an intensified time-stamping TPX3CAM.

**Fig. 3:**
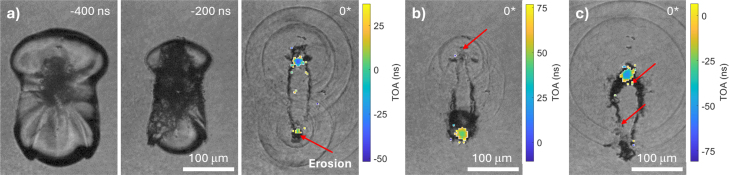
Bubble collapses in close proximity of a quartz surface (γ=0.11±0.01), leading to erosion and sonoluminescence. (a) High-speed camera frames leading to the bubble collapse with shock wave and sonoluminescence emission, as well as the separately detected positions of erosion marked with red arrows. The timing of the earlier illumination pulse in the frame closest to the collapse is a zero reference in relation to the ToA measurements (shown in overlay), denoted using a 0* sign. (b) and (c) High-speed camera frames closest to collapse for two different events at the same experimental setting, but with different outcomes compared to (a) due to significant statistical variations. Relative timings of collapses and sonoluminescence light time of arrival (ToA - from TPX3CAM, see text) are as following for each collapse spot: (a) Top: –30 ns & –35 ns; Bottom: –10 ns & 0 ns; (b) Top: 0 ns & –; Bottom: 40 ns & 40 ns; (c) Top: –60 ns & –50 ns; Bottom: 20 ns & –. The number pairs are always formed as “collapse timing & ToA”. The precision of collapse timing measurement is ±10ns, the precision of ToA ±5ns, and – signifies no data point available. The corresponding videos in normal-view (5 MHz framing rate) and side-view (144 kHz framing rate) are presented in Supplementary Movies 3–8.

Three distinct and typical phenomena distributions that occur during bubble collapse in close proximity of a surface are presented in Fig. 3 for the case of a quartz surface and γ=0.11±0.01. The ultra high-speed camera frames show the bubble at the moment closest to the collapse. We define the timing of the first illumination pulse in the frame showing the shock wave as a zero reference time for ToA measurements. When two shock wave fronts visualizations are captured, the zero time corresponds to the first exposure, i.e. capturing the inner shock wave. The double exposure improves time resolution, e.g., in Fig. 3b, where only one shock wave visualization is captured in the frame, shock wave emission must have occurred between the first and second exposures. For the three examples in Fig. 3, the experimental parameters remained unchanged, yet subtle differences originating from the laser-induced breakdown amplify during collapse, resulting in a bifurcation of the bubble dynamics approximately one microsecond before collapse. It was reported that small variations in the bubble shape and stand-off distance influence the shape of the resulting toroidal bubble and as a consequence its energy focusing geometry [41]. In all cases, small asymmetries lead to formation of an elongated torus-shaped bubble that exhibits two strong collapse spots, recognized as sources of the emitted shock waves. The shock waves are emitted from the two distant locations nearly simultaneously, e.g., in Fig. 3a the upper and lower part of the collapse happen within less than 20ns. In contrast, in Fig. 3b the lower part has collapsed first, while in Fig. 3c the upper part collapsed first. Yet, all three cases result in pits generated on the glass surface that are detected *post festum* by subtracting the images of the substrate recorded before and after each cavitation event. The locations of the erosion pits are indicated in overlay of the closest to collapse frame in Fig. 3a–c using red arrows. The arrows point to the locations of the detected erosion damage in that event, while damage itself is not visible in the presented frames.

The erosion patterns were found in three distinct regions around the bubble collapse spots: primarily directly below the bubble collapse spot, but to a smaller extent also along the torus-shaped shockwave-focusing region, and sometimes even outside the intense collapse region. Based on these distinct regions we define three types of erosion, being primary erosion, shock-wave focusing region erosion, and secondary erosion. The mechanism of the primary type of erosion has been identified in Reuter et al. [24] and explained as the final result of shock wave focusing on ductile materials. We assume that this mechanism also holds on brittle glass [19]. The two other types of erosion are weaker and significantly less common. An example of erosion found along the shock wave focusing region is shown for the lower red arrow in Fig. 3b. In certain events secondary erosion damage occurs on defects present from previous events, but is not marked on the Figure as it is not considered a side effect. However, an example of the eroded surface via the last mechanism are the three irregular spots in the quartz surface visible in Fig. 3a and c above the top collapse spot.

In addition to erosion detection and precise collapse time measurements, the spatially resolved light emission measurements obtained from the TPX3CAM are shown as an overlay of Fig. 3. The ToA measurements are color coded for a timespan of 85ns, with zero value matching the reference 0* time. We find light emission coming from spots either at the top, the bottom, or both collapses. The spatial centers of collapses and sonoluminescence overlap within the resolution of measurements, limited by the rather large pixel size of the TPX3CAM and its’ tendency to expose the neighboring pixels to the actual source. The light emission timing is measured with respect to the previously defined time zero, i.e., a positive ToA corresponds to light emission later than the first illumination pulse and negative ToA corresponds to earlier emission. The inherent measurement noise is reduced by gating the ToA events over an interval of 130 ns during postprocessing. This gate interval is centered around the known collapse time determined from the high-speed videos. The timings reveal that the time interval between the two collapse events is in the range of 20–40 ns and the sonoluminescence occurs simultaneously with the event of shock wave emission, within the margin of error. Detailed ToA and collapse timings are given in Fig. 3 caption.

### 3.3 Correlation of sonoluminescence, shock waves, and erosion

**Fig. 4:**
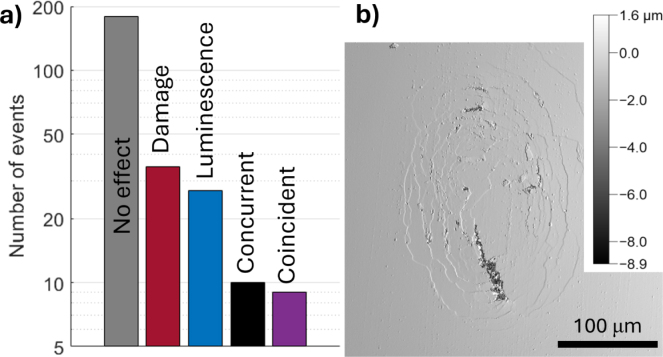
(a) Histogram of effect distribution for 130 bubble collapses exhibiting a total of 260 collapse spots, resolving concurrent and fully coincident sonoluminescence and erosion. The cavitation bubble with $R_{\text{max}}=0.48\pm 0.02\;\;\mathrm{mm}$ is positioned at γ=0.15 from the quartz surface. (b) Surface topography measurement of quartz after 80 events. Quartz surface is located at 0.0μm z-position.

**Fig. 5:**
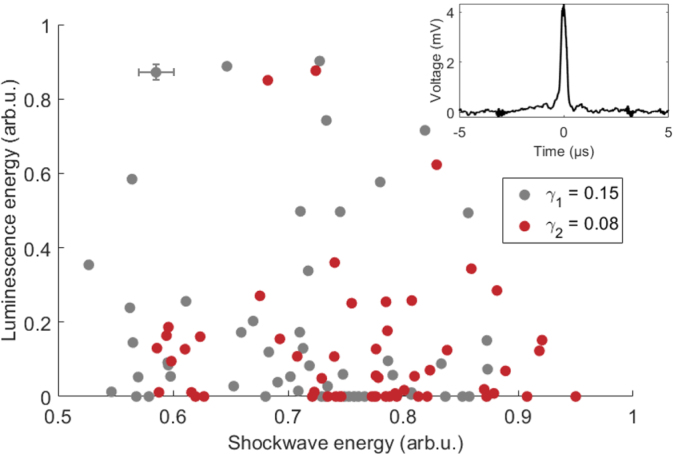
Graph of total sonoluminescence energy versus detected shock wave energy (both normalized) for experimental runs at $\gamma_{1}=0.15\pm 0.02$ and $\gamma_{2}=0.08$±0.01 on aluminum substrate. Every bubble collapse results in shock wave emission, but not necessarily sonoluminescence. The shock wave strength is not correlated to the luminescence energy. Inset shows a typical shock wave pressure signal measured by a needle-probe hydrophone and post-processed with low pass filtering.

An important observation from Fig. 3 is that not every collapse spot yields sonoluminescence or erosion, and likewise not every sonoluminescence event corresponds to observable erosion at the spot. Using a 6 mm thick quartz substrate, we analyze a series of 130 bubble collapses equaling 260 collapse spots at γ=0.15, where the strongest light emission was measured, see Fig. 2b. For each cavitation event, the presence and location of light and the presence and location of surface erosion are measured. Fig. 4a summarizes the effects observed at 260 collapse spots, split into two experiment runs with 50 and 80 bubble collapses at two separate locations on the sample, but at the same γ. The number of bubble collapses at each position was limited to avoid excessive damage to the sample surface. Fig. 4b shows a confocal surface scan of the quartz after the series of 80 bubble collapses. We find erosion patterns down to a depth of approx. 9 μm, which is a lower erosion rate compared to ductile metals [24], however, quartz is in comparison a significantly different materials in terms of elasticity and yield strength. Here, only erosive events corresponding to the shock wave focusing event at the collapse spots are considered, as the location of erosion and sonoluminescence are matched in this case. For sonoluminescence, the brighter spot was taken in the rare case of two light emission centers, signifying a higher energy focusing event similar to Fig. 3a, where top spot is significantly brighter. Aside from this exception, every detectable sonoluminescence emission source was taken into account to compile the data for both Fig. 3, Fig. 5. The latter plots two sonoluminescence energy distributions as a function of the pertaining shock wave energy.

Of the 130 bubble collapses exhibiting two collapse spots per event, documented in Fig. 4, we find a total of 54 erosion damage events and 46 sonoluminescence events. This means the probability for sonoluminescence is $p_{s}=\left(17.7\pm 2.4\right)\;\text{\%}$ and probability for erosion $p_{e}=\left(20.8\pm 2.5\right)\;\text{\%}$ per collapse spot, assuming a binomial distribution for each observable. In only 9 cases a coincidence occurs, causing light emission and erosion damage at the same collapse spot, with an additional 10 concurrent events observed, with erosion and sonoluminescence detection at the same bubble collapse but at separate collapse spots. If both events were to occur randomly and independently with the measured probabilities, the probability for a coincident event would be $p_{c}=p\left(e\cup s\right)=p_{e}\cdot p_{s}=\left(3.7\pm 0.9\right)\;\text{\%}$ resulting in 9.5±2.5 events in a sample size of 260 collapse spots. The uncertainty here is propagated from the input binomial distributions. While the near-boundary collapse creates conditions that lead to both phenomena, this finding points to the conclusion that erosive damage and sonoluminescence occur coincidentally under very similar energy focusing conditions but are not correlated. We thus further validate the previously identified mechanisms for single-bubble erosion that has been shown to be a consequence of shock wave focusing in similar conditions, while the sonoluminescence stems from extreme matter states reached inside the bubble [9], [31].

We further test the hypothesis if sonoluminescence and the strength of the shock wave emission are correlated. The pressure of the collapse shock waves is measured with a needle probe hydrophone (Müller-Platte) located about 3.5 mm from the laser focus. This distance is sufficiently far away not to affect the bubble dynamics while capturing clear pressure signal (shown in the inset of Fig. 5). Assuming a plane wave, the shock wave energy $E_{sw}$ is proportional to $E_{sw}\propto \int p^{2}\;dt\propto \int U^{2}\;dt$, where p is the shock wave pressure and U the voltage signal measured with the hydrophone. Only bubbles collapsing in a narrow temporal interval of $t_{c}=95\pm 5\;\mu s$ are considered to ensure a narrow distribution of bubble radii and therefore a narrow range of stand-off distances γ. Fig. 5 plots two sonoluminescence energy distributions, defined as the integrated luminescence intensity over all bright pixels, as a function of the shock wave acoustic energy. All collapses result in shock wave emission that has a normalized energy range between 0.5 and 1. Interestingly, light emission is observed across the whole range of acoustic emission energies with no clear trend in its probability. Many of the stronger shock waves are accompanied by weak or no light emission at all, and even the brightest sonoluminescence events are found across the range. Fig. 5 thus reveals no obvious correlation between the magnitudes of these two observables. A representative error bar is added to the top-left data point in Fig. 5 to highlight that the observed spread is not caused by measurement uncertainty.

These findings again indicate that the shock wave emission in the liquid and the heating of the gas may be spatially overlapping, but are the consequence of two different physical mechanisms. In other terms, sonoluminescence is an indicator that the condition for energy focusing occurs, yet the compression of the bubble interior and the mechanical loading of the nearby boundary are distinct from each other. A possible explanation why these two phenomena occurring on average at the same location are not correlated could be that erosion, although being triggered by the shock wave focusing, is caused by converging surface waves. We find that specific bubble shape development sequence occurs within the last microsecond before collapse. As a result, surface waves (Rayleigh waves) are likely excited continuously by the progressively collapsing toroidal bubble. Based on existing research, the weak acoustic waves generated at the beginning of the toroidal bubble collapse lead to intensified bubble collapse along the toroid. Due to this compression a shock wave is emitted and interacts with the solid boundary, where the Rayleigh waves are produced and focused similar as the shock waves in the liquid. Once the superpositioned tensile stresses overcome a threshold, a brittle failure of the glass occurs. Damage at a glass-water boundary due to surface waves has been observed in similar settings. In particular, radially outgoing surface waves [42] and inwards focusing surface waves [43]. The latter work utilized a ring like focusing geometry, where the Rayleigh waves arrived at the same focus as the acoustic wave yet around 10–20 ns earlier. In both experiments it was demonstrated that Rayleigh waves created from localized high pressures at the glass water interface can cause brittle failure of the glass. Still more work is needed to balance the importance of surface waves versus shock waves for cavitation erosion.

## 4 Conclusion

We observed, measured, and quantified cavitation bubble sonoluminescence for bubbles generated near a solid boundary and its relationship with erosion and emitted shock waves. Sonoluminescence was detected within a narrow parameter window of normalized stand-off distances 0.1<γ<0.3, i.e., in a range where no sonoluminescence has been reported previously. Within this range, the bubble shape is highly non-spherical during the entire oscillation. The collapsing bubble emits strong shock waves from two main collapse spots and causes cavitation erosion to the nearby surface at either or both spots. Individual bubble collapse event measurements reveal erosion and sonoluminescence emission are not correlated within the margins of statistical significance and may occur at different collapse spots, one phenomenon at a time, or entirely independently. Additionally, emitted shock wave energy was also not correlated with sonoluminescence. However, bubble shape and dynamics conditions for sonoluminescence directly correspond to conditions for strong cavitation erosion. The findings underscore previous research conclusions that shock wave focusing is responsible for erosion. In line with related recent works on cavitation erosion and the role of surface Rayleigh waves and shock waves in the fluid, we speculate that Rayleigh waves excited by the shock waves are responsible for the observed strong cavitation erosion. Further work is needed to study possible relations between sonoluminescence and more generalized strong collapses near surfaces. Conversely, the study demonstrates that even a non-spherical bubble collapse near a rigid boundary can generate sufficient volume compression for sonoluminescence.

## CRediT authorship contribution statement

**Jaka Mur:** Writing – original draft, Visualization, Methodology, Investigation, Formal analysis, Data curation, Conceptualization. **Rok Petkovsek:** Writing – review & editing, Writing – original draft, Supervision, Resources, Methodology, Investigation, Funding acquisition, Formal analysis, Conceptualization. **Claus-Dieter Ohl:** Writing – review & editing, Writing – original draft, Supervision, Resources, Methodology, Investigation, Funding acquisition, Formal analysis, Conceptualization.

## Declaration of competing interest

The authors declare that they have no known competing financial interests or personal relationships that could have appeared to influence the work reported in this paper.

## Data Availability

The data that support the findings of this study are available from the corresponding author upon reasonable request.

## Appendix A Supplementary data

Supplementary Movie 1 1 MHz video of bubble dynamics in side-view for event in Fig. 2a. Scale bar equals to 200 μm. Supplementary Movie 1

Supplementary Movie 2 5 MHz video of bubble dynamics around collapse in side-view for an alternate event. Scale bar equals to 200 μm. Supplementary Movie 2

Supplementary Movie 3 5 MHz video of bubble dynamics around collapse in normal-view for event in Fig. 3a. Scale bar equals to 100 μm. Supplementary Movie 3

Supplementary Movie 4 Complementary 144 kHz side-view video of bubble dynamics for event in Fig. 3a. Scale bar equals to 500 μm. Timestamps use μs. Supplementary Movie 4

Supplementary Movie 5 5 MHz video of bubble dynamics around collapse in normal-view for event in Fig. 3b. Scale bar equals to 100 μm. Supplementary Movie 5

Supplementary Movie 6 Complementary 144 kHz side-view video of bubble dynamics for event in Fig. 3b. Scale bar equals to 500 μm. Timestamps use μs. Supplementary Movie 6

Supplementary Movie 7 5 MHz video of bubble dynamics around collapse in normal-view for event in Fig. 3c. Scale bar equals to 100 μm. Supplementary Movie 7

Supplementary Movie 8 Complementary 144 kHz side-view video of bubble dynamics for event in Fig. 3c. Scale bar equals to 500 μm. Timestamps use μs. Supplementary Movie 8
